# Alternative alignment clinical study: study protocol for a prospective, randomised, controlled study of the outcomes and cost effectiveness of triathlon CR knee replacement system: traditional philosophy versus alternative alignment philosophy

**DOI:** 10.1186/s13018-025-06551-z

**Published:** 2025-12-05

**Authors:** Jonathon Sheen, Wahid Abdul, Ciara Corroon, Ben Waterson, Andy Toms

**Affiliations:** 1 University Hospitals Plymouth and University of Exeter, Exeter, UK; 2 Princess Elizabeth Orthopaedic Centre, Royal Devon and Exeter Hospital, Exeter, UK; 3 University of Plymouth, Plymouth, UK

## Abstract

**Background:**

Despite being one of the most successful orthopaedic operation, a number of patients remain dissatisfied after TKR surgery. There has been a recent move by some to change surgical philosophy to aim to replicate the alignment of the patient’s own original knee—known as kinematic or alternative alignment. The purpose of this study is identify if using the alternative alignment technique can lead to significantly improved outcomes when comparted to the traditional alignment technique.

**Methods:**

A single centre, randomised control trial, powered to recruit 126 patients (63 in each arm). The primary aim is to identify if the group undergoing TKR using the alternative alignment technique have a significantly improved functional outcome at 1 year when assessed by the minimal clinically important difference of at least 5 points in the Oxford knee score by comparison to the group using traditional alignment technique. Various other secondary outcomes are set out in the main text.

**Discussion:**

A study examining short-, medium- and longer-term outcomes of alternative alignment is needed to see if outcomes for patients can be improved. This study has the potential to be of great importance in contributing to this question. Limb alignment has focussed on the need to achieve the zero-degree goal of hip, knee and ankle alignment. To achieve real 3D more natural alignment, surgeons have to accept this goal may not be correct. There is a need to achieve pre-arthritic/pre-morbid alignment and to evaluate if this can unlock the potential for improved patient outcomes. A prospective, randomised study with multiple outcome measures can deliver a major contribution to the knowledge base of this subject and influence surgical practice with the aim of improving patient outcomes and satisfaction.

**Trial registration:**

The study was approved by the Wales Research Ethics Committee (IRAS project ID: 225871), and was registered with clinicaltrials.gov (Identifier NCT03196011).

**Supplementary Information:**

The online version contains supplementary material available at 10.1186/s13018-025-06551-z.

## Introduction

Despite being one of the most successful orthopaedic operation, a number of patients remain dissatisfied after TKR surgery. In a study of 253 patients, 1 year after TKR, Noble et al. [1] reported 11% of patients were neutral in their satisfaction after their surgery and 14% reported that they were either dissatisfied (3%) or very dissatisfied (11%). Reasons for this dissatisfaction are not always clear.

Various changes to surgical technique have tried to address this over the years. These include computer assisted navigation and patient specific knee replacement parts. However, whilst computer assisted orthopaedic surgery seems to improve accurate component positioning, the clinical significance of this remains to be proven [2].

More recently, attention has focussed on the alignment of the leg. Classic mechanical alignment in TKR is meant to co-align the prosthetic components to the mechanical axes of the femur and tibia and restore neutral overall limb alignment [3]. Traditionally, surgeons have believed that implant durability is maximized when postoperative limb alignment is corrected to 0° ± 3° relative to the mechanical axis [4]. When implanting the tibial component of a TKR, the surgeon will try and put the device perpendicular to the tibial mechanical axis. This is done to try and make sure the stresses on the new knee are equally placed on both the medial and lateral sides of the joint to try and prevent asymmetrical or accelerated wear on one side of the prosthesis. However, Bellemans et al. [5] examined 250 asymptomatic volunteers and identified that 32% of men and 17% of women have a varus deformity of the knee. Therefore, for many people, placement of the tibial component perpendicular to the floor may result in the alignment of the leg changing considerably, putting new strains on the soft tissues around the knee itself as well as altering the biomechanics of the adjacent ankle and hip joints with the potential to generate soft tissue strain in these areas also. It is possible that this alignment change is a major contributing factor to the dissatisfaction of some patients with TKR.

Concerns that a failure to achieve “correct” positioning of the TKR components may adversely affect the survivorship of the artificial joint may not be well founded. Bonner et al. [6] reported upon the survivorship 501 TKRs after 15 years of follow up. 396 implants were within 3° of the mechanical axis and 105 were not. Whilst this latter group may have been expected to have performed inferiorly, the results showed no statistically significant difference in the survivorship of the 2 groups when revision for aseptic loosening was used as the end point for the analysis. Similarly, Parratte et al. [4] reviewed 398 TKR cases in whom 292 had post-operative limb alignment within 0°–3° of the mechanical axis and 106 were outside of this. They concluded that a postoperative mechanical axis of 0° ± 3° did not improve the fifteen-year implant survival rate.

Surgeons are therefore now questioning the old philosophy of alignment [7]. There has been a move by some to change their surgical practice, and instead to try and put the knee replacement parts in place in a way that replicates the alignment of the patient’s own original knee. This is known as natural, kinematic or alternative alignment. Several studies have been undertaken that look at this alternative alignment, and importantly, they have not shown any loss of satisfaction with results of knee replacement, and indeed are showing some signs that in the short term, function of the knee may be better with the new technique [8, 9]. A meta-analysis by Migliorini et al. [10], which took 30 studies into account, found no statistically or clinically significant difference in PROMs and revision rate between mechanical and kinematic alignment.

Waterson et al. [8] reported one year follow up data in 71 patients undergoing TKR randomised to either kinematic (n = 36) or mechanical alignment (n = 35). Kinematically aligned TKRs were reported to have comparable short-term results to mechanically aligned ones with no significant differences in function one year post-operatively. Interestingly, in this work, there was a greater improvement in the mean American Knee Society Score (AKSS) in the kinematically aligned group at six weeks when compared with the mechanically aligned group (p = 0.05). The measurements of peak torque in the quadriceps were also significantly better in the kinematically aligned group at six weeks and three months (*p* = 0.003 and *p* = 0.02 respectively) by comparison to the mechanically aligned group.

Mizner et al. [9] reported deficits in quadriceps force of 30–40% in patients for up to a year after TKR by comparison to age matched subjects. They describe reduction of quadriceps muscle performance has been correlated with fall risk, walking speed, speed and quality of sit-to-stand transfers, and performance during stair climbing in individuals older than 60 years of age. Such functional deficits can impact significantly on quality of life and this functional reduction could be a contributory factor to the dissatisfaction with TKR reported by some patients. Bade et al. [11] identified similar reductions in quadriceps strength after TKR at 1 and 3 months post-op that took 6 months to recover to pre-operative levels. If Waterson’s work on kinematic versus mechanical alignment shows improvements in early quadriceps function, then it is important to see if this can be progressed further and translated into improved function and satisfaction after TKR.

Dossett et al. [12] reported the results of their work and evaluated an alternative alignment method (kinematic alignment with patient-specific cutting guides) compared to the alignment results with mechanical alignment with conventional instruments. In this work, the authors point out that because a mechanically aligned TKR strives to correct limb alignment to a straight line, the kinematics of the knee can be altered. Two of their secondary outcome measures showed results in favour of the kinematically aligned knee: the Oxford score (OKS) (0 is best and 48 worst) was 8 in the kinematically aligned group and 15 in the mechanically aligned group, a difference of 7 points, which was significant (*p* = 0.001). The range of knee flexion was 120° in the kinematically aligned group and 115° in the mechanically aligned group, a difference of 5°, which was significant (*p* = 0.043).

In the short term at least, it would appear that alternative or kinematic alignment of TKR components has the potential to deliver improvements in patient generated outcomes, quadriceps torque and flexibility of the knee which in theory could increase the number of patients satisfied with TKR.

A study examining short-, medium- and longer-term outcomes of alternative alignment, with a contemporary TKR design is needed to see if outcomes for patients can be improved. This study has the potential to be of great importance in contributing to this question. Limb alignment has focussed on the need to achieve the zero-degree goal of hip, knee and ankle alignment. To achieve real 3D more natural alignment, surgeons have to accept this goal may not be correct. There is a need to achieve pre-arthritic/pre-morbid alignment and to evaluate if this can unlock the potential for improved patient outcomes. A prospective, randomised study with multiple outcome measures can deliver a major contribution to the knowledge base of this subject and influence surgical practice with the aim of improving patient outcomes and satisfaction.

## Study aims and objectives

### Primary aim

To identify if the group undergoing TKR using the alternative alignment technique have a significantly improved functional outcome at 1 year when assessed by the minimal clinically important difference (MCID) of at least 5 points in the Oxford Knee Score (OKS) by comparison to the group using traditional alignment technique.

### Secondary aims

To identify if the use of the alternative alignment technique when performing TKR results in:

- A faster post-operative recovery assessed by length of stay (LOS) in hospital
- Improved short term function when assessed by peak quadriceps muscle torque measured by myometer
- Lower hospital costs as a result of reduced LOS
- Faster increase in hamstrings muscle mass/torque measured by myometer
- Enhanced improvement in patient outcome scores (Knee Osteo-arthritis Outcome Score (KOOS), Oxford Knee Score (OKS), EQ5D) at short-, mid- and long-term timeframes
- Enhanced improvement in the results of the Exeter Functional Lab protocol
- Enhanced activity assessment, as measured by the Exeter University Department of Sport Science using an accelerometer in a subset of the first 40 participants
- Enhanced positioning of the implants, as assessed by a CT scan rotational profile assessment
- Enhanced range of knee flexion
- Any difference in wear characteristics of the prosthesis
- Any significant differences in component positioning
- Any significant differences in limb alignment
- Any significant changes in 5 gait parameters as measured by the Instride Accelerometer
- Any significant difference in the Self-Administered Patient Satisfaction Scale (SAPS)

by comparison to the TKR performed with traditional instruments.

We will achieve these aims by:

- Enrolling 126 patients into this study over a period of 5 years
- Carrying out a prospective randomised study looking at the subject matter over a period of 11 years (5 years recruitment; 5 years follow up; 1 year data analysis and write up
- Analysing patients’ functional status pre- and post-surgery using patient generated questionnaires and data from the Exeter functional lab
- Analysing patients’ post-operative radiological results from the plain X-rays to look at limb alignment, component position and wear
- Analysing the TKR component position on the pre and post–operative CT scans
- Collecting length of stay data taken from the patient administration system
- Collating all of the data from the study into a spread sheet for analysis by a professional statistician

## Methodology

### Study design

This is a prospective, single-centre, partially blinded, randomised controlled trial conducted at the Exeter Knee Reconstruction Unit (EKRU), Royal Devon and Exeter (RD + E) Hospital. A total of 126 patients undergoing primary total knee replacement (TKR) will be randomised (1:1) to receive implantation using either traditional mechanical alignment or an alternative alignment philosophy. Randomisation will be performed using a computer-generated sequence, with allocation concealed in sealed opaque envelopes.

Blinding will be maintained for the patient, all outcome assessors, radiologists, physiotherapists, and university staff analysing accelerometer data. Blinding of these individuals will be ensured by not revealing the treatment arm to the outcome assessors, radiologists, physiotherapists and data analysts; and keeping mention of treatment arm to the operation note alone. The operating surgeon cannot be blinded due to the nature of the intervention. Follow-up assessments will be conducted at 6 weeks, 3 months, 6 months, 1 year, 2 years, and 5 years post-operatively.

The primary outcome is functional improvement at 12 months measured using the Oxford Knee Score (OKS). Secondary outcomes include muscle strength, implant positioning, radiological alignment, activity levels, patient-reported outcomes (KOOS, EQ-5D, FKS, UCLA, SF-36), range of motion, prosthesis wear, and healthcare utilisation.

### Identifying potential study participants

Patients will be identified as potential participants in one of two ways. Firstly, by their surgeon when they are initially listed for TKR surgery at their out-patient consultation at the RD + E. The surgeon will outline the study, provide the patient with the study patient information leaflet and a copy of the study consent form and ask permission from the patient to allow one of the research team to contact them to discuss the study further. Alternatively, potential participants will be identified from the current surgical waiting list as suitable for inclusion. In this latter case, their surgeon will write to the patient requesting permission for one of the research team to contact them by telephone to discuss the study and to enquire if the patient would enrol. A patient information leaflet about the study will accompany this letter.

The patient will have an opportunity to provisionally agree or decline enrolment during this telephone call.

The below table (Table 1), shows the enrolment, intervention and assessment timings, in accordance with the SPIRIT reporting guidelines. [13]

**Table 1:** Schedule of enrolment, intervention, and assessments

	Study period								
	Enrolment	Allocation	Surgery		Post-surgery				Close-out
TIMEPOINT**			0	6 weeks	3 months	6 months	1 year	2 years	5 years
ENROLMENT:									
Eligibility screen	X								
Informed consent	X								
Allocation		X							
INTERVENTION:									
Surgery			X						
ASSESSMENTS:									
CT	X			X					
X-rays	X			X					X
Questionnaires	X			X	X	X	X	X	X
Functional Assessments	X			X	X	X	X	X	X

**Fig. 1:**
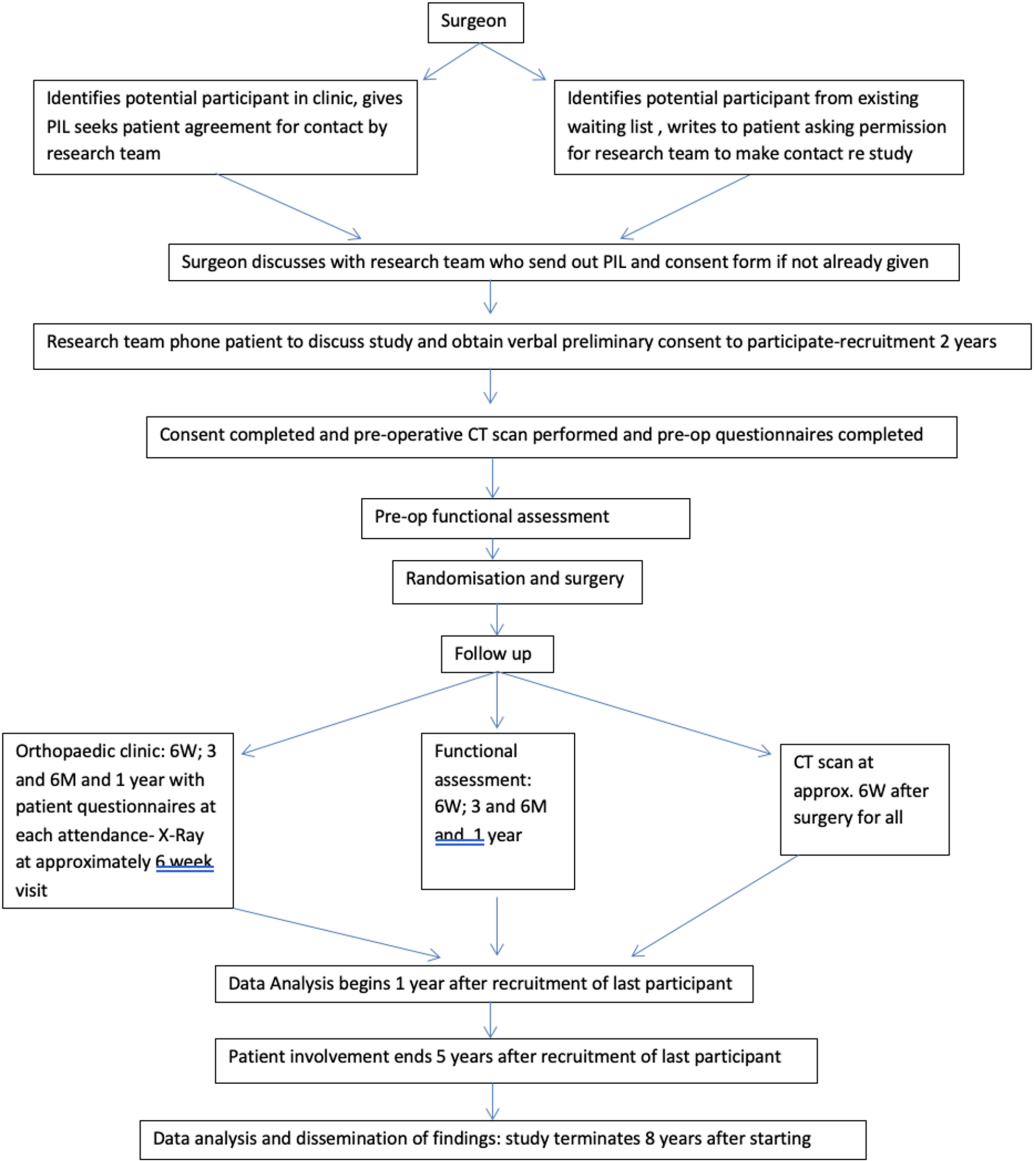
Participant timeline

The below figure (Fig. 1) shows a flow diagram of the participant timeline.

Inclusion criteria:

- Patients undergoing primary TKR at the RD + E Hospital
- Patients must have completed a consent form for the study
- Patients must be prepared to comply with the pre- and post-operative investigations, rehabilitation, attendance schedule and questionnaire schedule of the study
- Patient in whom any varus deformity present is < 20°
- The diagnosis is of tricompartmental osteoarthritis of the knee
- Patient has primary diagnosis of Non-Inflammatory Degenerative Joint Disease (NIDJD).
- Body Mass Index (BMI) < 40
- Aged ≥ 50 years at time of surgery

Exclusion criteria:

- Refusal to consent to the study
- If the knee for surgery has a fixed flexion deformity ≥ 15°- this will be assessed by a lateral “heel-hang” X-ray of the knee for surgery
- If the knee for surgery has a varus deformity ≥ 20°
- If the knee for surgery has a valgus deformity i.e. hip/knee/ankle alignment angle < 0°
- Pre-op Oxford Knee Score < 8
- Pre-op knee flexion ability < 90°
- Any patient whose post-operative recovery or ability to comply with the post-operative rehabilitation and assessment schedules is compromised by known existing other medical conditions
- Pregnancy
- Prisoners
- A patient known to have substance abuse or psychological disorders that could interfere with their ability to comply with the post-operative rehabilitation and assessment schedules
- Patients unable to read or understand the patient information leaflet and consent form
- BMI ≥ 40
- Patient has an active or suspected latent infection in or about the affected knee joint at time of study device implantation.
- Patient has received any orthopaedic surgical intervention to the lower extremities within the past year or is expected to require any orthopaedic surgical intervention to the lower extremities, other than the TKR to be enrolled in this study, within the next year.
- Patient requires bilateral total knee replacements, or has a history of unsuccessful contralateral partial or total knee replacement.
- Patient has chronic heart failure (NYHA Stage ≥ 2)
- Patient has a neuromuscular or neurosensory deficiency, which limits the ability to evaluate the safety and efficacy of the device.
- Patient is diagnosed with a systemic disease (e.g. Lupus Erythematosus) or a metabolic disorder (e.g. Paget’s disease) leading to progressive bone deterioration.
- Patient is immunologically suppressed or receiving steroids in excess of normal physiological requirements (e.g. > 30 days).
- Patient requires revision surgery of a previously implanted total knee replacement or knee fusion to the affected joint.

### Sample size

126 patients (63 per arm). The sample size was determined using the primary outcome measure, OKS measured at 12-month follow-up, which has an MCID of 5 units [14]. To detect a between group mean difference of 5 units, with 90% power and significance threshold of 0.05, using a standard deviation in both groups of 7.6, requires 50 participants per group. Thus, accounting for drop-outs, we aimed to recruit an additional 13 patients to each arm, bringing the total to 126 patients. For a completed SPIRIT checklist, see the additional file. [13]

### Obtaining participant consent/collection of pre-operative data

If the potential participants have given verbal agreement to enrol in the study, we will obtain written consent of participation when they attend the hospital for the pre-operative CT scan. The patient will meet one of the research team before the CT scan appointment in the out-patient orthopaedic appointment area to sign a study specific Ethics Committee approved study consent form. They will also complete the pre-operative data will be collected including: demographics and medical history, KOOS, OKS, the EQ5D score, the Forgotten Knee Score and the SF36, the UCLA activity score, pain measurements by Visual Analogue Scale (VAS) during rest and activity and detail of the use of pain medication. The research team member will perform an assessment of the participants overall joint flexibility using the Beighton scale. An assessment of pre-operative anxiety and depression will be made via the Hospital Anxiety and Depression Scale.

On the study consent form, we will ask for the participant’s agreement to contact their GP to inform them of inclusion in the study.

We will ask for specific patient consent to send personable identifiable information (patient name, date of birth and hospital number) as well as CT scan and X-ray images to LifeIMAGE in the United States (who act as a pass-through server to the Stryker segmentation team). We will also ask for the same permission for the scans to be passed to Stryker US via LifeIMAGE for the purpose of unique operation detail planning.

LifeIMAGE and Stryker US are the only companies able to provide the individually tailored surgical plan from the CT and X-ray data for use with the MAKO system.

3 copies of written consent will be obtained- one original and 2 photocopies. The original will be kept by the research team in the patient’s case record file (CRF), one will be returned to the patient and one will be placed in the medical notes.

Routine care for these patients from their clinic appointment will have already included X-ray of the affected knee, full leg alignment X-ray, and an assessment of their active and passive range of motion of the knee.

All information collected pre-operatively will be used to quantify the sample population and compare post-operative progression.

### Randomisation

This will be a randomised, prospective, blinded study with 2 arms of 63 patients each. The first arm will receive a Triathlon knee replacement device which is implanted using the traditional alignment philosophy in which the tibial cut is made perpendicular to the tibial anatomical axis. The second arm will receive a Triathlon TKR using the alternative alignment philosophy which aims to replicate the natural alignment of the knee.

Patients who agree to enter the trial will be divided at random to enter the different treatment arms of the study. The randomisation schedule will be determined using an on-line random assignment sequence generator (www.graphpad.com/quickcalcs/randomize1.cfm) and will be kept and controlled by the study co-ordinator, and the surgeons will not have access to this schedule. 126 patients will be included. Their randomisation assignment will be transcribed onto paper, and placed inside a sealed opaque envelope. The study co-ordinator or research nurse will inform the operating surgeon of the assignment of each patient at the weekly surgical planning meeting.

Any deviation from the assigned treatment group by a surgeon will be reported as a deviation from protocol and the case will be terminated from the study.

### Study blinding

The following people will be blinded as to which arm of the study the patient is allocated to:

- The patient
- The functional assessor in the physiotherapy department
- The University staff who assess the activity levels identified by the accelerometer
- The physiotherapist providing post-operative physiotherapy
- The radiologist reporting the CT scans
- The 2 surgeons reporting the X-ray findings—no surgeon will report on his or her own patients

### Timescale

EKRU carry out 25 TKR procedures per month. We understand that studies have, on average, 54% of potential participants who choose not to be part of the research. Using this figure, we believe it is reasonable to recruit 11 cases per month. However, we will allow for 60 months to achieve our sample size of 126 bearing in mind the suspension of surgery that occurred between 2020 and 2022.

Each patient will remain part of the study for approximately 5 years.

Total duration of the study will be 11 years. This comprises 5 years to recruit participants, 5 years of follow up and data collection and one further year for data collation, cleansing and evaluation, statistical analysis and writing up of the study findings.

### Pre-operative radiological assessment

In addition to routine care, all study participants will undergo (1) a pre-operative CT scan of the affected leg which will be a spiral CT through the hip, knee and ankle, and (2) a whole leg antero-posterior radiograph standing on one leg (the leg about to have a TKR and involved in the study) and (3) a lateral “heel-hang” X-ray of the knee for surgery taken on a 17-inch X-ray plate.

### Pre-operative functional assessment

In addition to routine care, all study participants will attend the Functional Lab in the physiotherapy department at the RD + E Hospital for an assessment of their functional ability, using the Exeter Functional lab protocol and the InStride Accelerometer. The InStride accelerometer is a device used to measure speed of walking, stride length, equality of weight-bearing in walking. It is an app on a mobile phone. The patient clips the phone to their belt, walks 40 paces and the device measures the gait pattern and features. This is in addition to routine care.

In the subset of the first 40 participants selected to wear an activity monitor (accelerometer) physical activity will be assessed for 21 continuous days using a GENEActiv (ActivInsights Ltd, Kimbolton, UK) tri-axial accelerometer, measuring 43 mm × 40 mm × 13 mm. The GENEActiv will be attached to a polyurethane strap, and worn at the left wrist, like a watch. It can measure between ± 8 g at a rate of up to 100 Hz. During the proposed study, data will be collected at a rate of 20.0 Hz to permit 21 days of continuous monitoring.

Details of participants selected for physical activity monitoring will be sent to the research team in the Department of Sport and Health Sciences at the University of Exeter, St Luke’s campus. They will send out accelerometers at each time point with prepaid return envelopes along with a participant guide for wearing the accelerometer and a short log for recording when the device was fitted and removed. Participants will also be asked to record the time they go to bed and get up each day to assist with data analysis.

### Intra-operative assessment

Based on pre-operative CT scans, the MAKO system generates a 3D computer image of a patient’s knee joint.

This unique 3D computer image is used by the surgeons to determine how much bone to remove and where to place the knee replacement implants.

### Post-operative follow up

Participants will be reviewed at 6 weeks, 3 and 6 months, 1, 2 and 5 years post-operatively. The 3 and 6 month appointments are in addition to routine care. The 1, 2 and 5 year follow ups are usually done by postal questionnaire but will require hospital attendance in Exeter for the purpose of the study as they will undergo a repeat of the functional testing at each time interval. The 6 post-operative visits for functional testing are in addition to routine care.

The cohort of 40 patients who used the activity monitoring accelerometer provided by the Department of Sports Science at Exeter University pre-operatively will be given another device whilst an in-patient in hospital. This will be worn continuously for a further 21 days before being returned to the University in a stamped addressed envelope that has been provided for that purpose. This will accurately log their post-operative activity levels. The cohort of 40 patients who used the activity monitoring accelerometer provided by the Department of Sports Science at Exeter University pre-operatively will be posted another device 3 months after surgery. This will be worn continuously for a further 21 days before being returned to the University in a stamped addressed envelope that has been provided for that purpose. This will accurately log their 3-month post-surgery activity levels.

### Post-operative radiological assessment

Participants will undergo X-ray examination of the knee post-operatively whilst in hospital and then at 6 weeks, 2 and 5 years.

Participants will undergo one post-op CT scan examination of the affected leg at approximately 6 weeks after surgery which will be a spiral CT through the hip, knee and ankle and a repeat of the whole leg antero-posterior radiograph standing on one leg (the leg that has just had a TKR and is involved in the study) at 6 weeks post-operatively. This is in addition to routine care.

### Assessment of outcome

We will assess the results in a number of different ways including validated patient generated outcome questionnaires, functional assessment (strength, flexibility and the ability to carry out everyday tasks such as rising from a chair, climbing stairs, walking on rough ground), measurement of activity levels and radiological assessment of alignment.

### Primary outcome measure

- The assessment of difference in function between the 2 study groups as measured by the OKS. The MCID for this outcome measure is 5 points. We will assess this at 1 year post surgery.

### Secondary outcome measures

- Assessment of peak quadriceps muscle torque measured by myometer at 6 weeks, 3 and 6 months and 1, 2 and 5 years post op.
- Measurement of length of stay (LOS) in hospital
- Measurement of lower hospital costs as a result of reduced LOS assessed between the arms of the study
- Assessment of hamstrings muscle mass/torque measured by myometer at 6 weeks, 3 and 6 months and 1, 2 and 5 years post-op.
- Patient generated outcome scores (KOOS, EQ5D, the Forgotten Knee Score (FKS), UCLA activity score, SF36 at 6 weeks, 6 months and 1, 2 and 5 years post-op.
- Assessment of function using the Exeter Functional lab protocol at 6 weeks, 3 months, 6 months, 1, 2 and 5 years post-op.
- Assessment of function using the Instride Accelerometer
- Assessment of activity levels as measured by an accelerometer used for 21 days at the pre-surgery, post-surgery and 3 months after surgery time frames.
- Self-Assessment of Patient Satisfaction via SAPS questionnaire at 6 weeks, 6 months, 1, 2 and 5 years post op.
- Assessment of positioning of the implants and of joint line position by a comparison of pre-op and 6 weeks post op CT scans.
- Measurement of range of movement of the knee at 6 weeks, 3 months, 1, 2 and 5 years post-op.
- Assessment of wear of the prosthesis on X-ray at 6 weeks, 2 and 5 years.
- Any significant differences in component positioning measured on the full leg alignment X-ray at 6 weeks.
- Any significant differences in limb alignment measured on the full leg alignment X-rays at 6 weeks

### Surgery

The standard MAKO TKR protocol will be followed with separate algorithms for the 2 alignment philosophies. A template operation note has been included as Appendix 1. Alignment of the components which will be decided upon in the randomisation schedule for use of the traditional mechanical alignment or the alternative alignment technique. The 4 surgeons involved in this study are all experienced TKR surgeons.

All components will be cemented in situ. All patients receive the Triathlon CR Implant with an X3 polyethylene insert. All patellae will be resurfaced.

After surgery, patients will be discharged home allowed to fully weight-bear as comfort allows and with walking aids for use for a 6-week period if required. All patients will have been instructed in an exercise regime whilst in hospital which they will continue after discharge and post-operative out-patient physiotherapy will be provided on an as needed basis. The physiotherapy treatment (type and frequency) will be decided upon by the individual physiotherapist seeing each patient. This reflects the care given to TKR patients in a part rural region and mirrors the care available which is given on an as needed basis.

### Data collation

To be undertaken by the clinician undertaking the specific procedure concerned.

Patrick Hourigan or the research nurses attached to the RD + E Hospital R + D department will collect the pre-op consent, the pre- and post-op PROMs. Data will be entered on to the study database by them the accuracy of entry double checked by them i.e. one person enters the data and a second checks its accuracy.

RD + E Hospital physiotherapists will be responsible for collection of the Functional Assessment data.

Colleagues at the University of Exeter will be responsible for collating the accelerometer data on the participants who use the 21-day device. Data returned via the post will be downloaded using GENEActiv PC software version 1.4 and analysed using the GGIR software package for R (cran.r-project.org). Data Time spent in different physical activity intensities will be estimated using published accelerometer cut-points.

The 4 surgeons will be responsible for clinic data and communications, operation note details, reporting on X-ray findings- all of these to be on the hospital Clinical Documentation Management system.

CT scan findings will be reported as with any CT scan undertaken within our hospital. The data from the CT scan and X-ray images will be used by Stryker US client application to direct the surgeon via the MAKO system in how to perform the bony cuts on each participant.

Data from the InStride accelerometer will be collected separately and using only the patient’s unique study number for identification. The data is captured on an I-phone specifically purchased for this purpose by our Unit. The accelerometer includes a Clinician Mode accessed through a secure authorization code that allows the capture of walk patterns in a clinical setting with the walk data stored only on the app portal. In this mode no data is stored on the Smartphone used to capture the walk.

Similarly, secure clinician access to the InStride Web portal allows download of the unprocessed triaxial accelerometer data in addition to viewing the users five walk parameters.

All other data will be stored on RD + E NHSFT secure computers. Data will be entered into an Excel spreadsheet for later transfer into a statistical package such as SPSS v24, for analysis. All patient identifiers will be removed. The study data will be entered by a data clerk and double checked by either the study-co-ordinator or one of the research nurses. At the end of the study, when all analysis of the data has been completed, the study data will be archived as per our sponsor’s archiving SOP.

## Statistical analysis

Independent statistical analysis of the results will be undertaken by an experienced statistician from the R + D department within our hospital.

All outcomes will be reported descriptively by group, with continuous outcomes reported as mean (standard deviation) or median (interquartile range).

All continuous outcome measures, including the primary outcome (OKS) will be analysed using linear regression modelling with adjustment for randomisation variables (surgeon, severity based on baseline OKS, and age (below 70, 70 and above), and baseline measurement). EQ5D will be analysed using the equivalent analyses with Tobit regression. Between group comparisons will be reported using confidence intervals and p-values.

For continuous measures recorded at multiple time points, hierarchical regression models will be used to assess the interaction between intervention and timepoint, with the interaction effect reported as a global p-value.

All analyses will be reported using the intention to treat principle. The primary analyses will include complete case data only. The amount of missing data will be reported for all outcome variables. In the event of substantive missing data (more than 5%), a secondary analysis will be performed including observed data and missing data imputed using multiple chained equations.

## Dissemination of results

The participants will be informed in writing of the results if they so desire. The Research and Development Office at the RD + E will be informed of the results.

We plan to present the results both nationally and internationally at knee surgery conferences and to publish the results in a peer reviewed journal.

### Impact of research

The impact of this study as yet is unknown. If we identify a clear advantage of one study arm over the others, then it is likely that this technique will become our standard TKR procedure- with the aim of providing patients with the best clinical and functional results after their TKR surgery. Similarly, if a clear difference is found in one study arm, one would hope that presentation of the results to colleagues at other institutions will result in better long-term results for their patients.

In the event that no technique proves superior to the others, then this will provide surgeons with the reassurance that no matter what their preferred technique, their patient is not being disadvantaged.

If one arm of the study out performs the other in clinical and functional results, there may also be cost differences between the arms of the study which could result in cost savings for the NHS.

## Patient involvement

We have consulted with patients on the feasibility of participating in the study at all and in particular about the demands of attending the appointments for functional assessment and CT scans. The study was discussed at our patient research panel. This panel is a group of patients who have undergone knee replacement surgery in our Unit and who are therefore, well placed to comment on the practicalities of study involvement, as well as on the appropriateness and clarity of the patient information leaflet. Attendance at all time frames is critical for completion of the secondary measure functional outcome and it was stressed by our patient representatives that the patients need to be fully informed of the commitment required should they agree to take part. Similarly, it was recommended that the extra commitment for CT scan appointments be highlighted.

We hold bi-annual meetings of the patient research panel and if issues arise when running the study that require further patient involvement, we will take them to the panel for consultation. If a meeting is not due for some time, we will contact a panel member personally for their advice.

When we consulted on the design and wording of the patient information leaflet and the consent form, various changes were recommended and implemented.

### Risks/monitoring adverse events/stopping the study

A log of adverse events, serious adverse events, adverse device effects and serious adverse device effects will be kept by the study co-ordinator. Any such events occurring in the immediate peri- and post-operative events will be reported to the consultant surgeon in charge of the case who will contact the study sponsor within the time frames as specified by them. Similarly, any such adverse events may be identified in clinic and again, the surgeon concerned should report these back to the study coordinator to be logged, and the sponsor will be informed.

It is possible that adverse events will occur that are dealt with either by the A + E department, the patient’s GP or other hospitals. These may not be readily apparent to the research team. We will specifically ask participants to inform their surgeon if any complications or adverse events occur and similarly ask the surgeons to specifically ask their patients for this information at each clinic attendance.

A meeting of the research team will be held on a 3-monthly basis to discuss progress of the study, any specific problems and to consider whether there are any justifications for stopping the study. As both techniques being studied of performing rTKR are already in use, we do not anticipate identifying an unexpected complication that would necessitation an early termination of the study. During these meetings, it will also be assessed if any deviations to the protocol have occurred, and if any amendments to the protocol are required.

## Supplementary Information

Below is the link to the electronic supplementary material.

Supplementary Material 1

## Declarations

### Confidentiality

All of the research staff will be GCP trained. All NHS staff involved are bound by patient confidentiality requirements of the NHS. The study participants GP will be informed that their patient is involved in the research- particularly as the patient may wish to discuss their involvement in the research with their GP. With the exception of the information collected (1) through the Instride Accelerometer, (2) the 21 day accelerometer data collated by the University of Exeter, and (3) the surgical planning data, all of the data associated with the study will be kept electronically on the hard drives of computers within the Exeter Knee Reconstruction Unit (EKRU). It will be stored on encrypted and password protected hospital computers. The data will only be accessible by members of the research team and by the R + D department at the RD + E Hospital who will require access to the data to ensure compliance with the protocol and that we are meeting all study regulatory requirements. Re the InStride accelerometer: For clinical patients, they will first access the InStride app and web portal with their clinician (physiotherapist or surgeon) and be directly on boarded to InStride. The onboarding includes providing their unique study number as the only means of identification. This contains no personal data. It is entered by the clinician into the InStride web portal. The InStride user provides their unique study number to an authorized clinician for entry into the InStride web portal to generate a specific user profile. The authorized clinician generates a token (unique code) for that user profile that is then entered into the InStride app. The token provides the secure match between the user profile and the Smartphone. The user’s walk pattern is compared only to their own baseline (set as a 30 or 40 m straight line walk in the presence of their clinician) and does not mingle with any other users’ patterns. InStride is approved for use at Nova Scotia Health Authority and has passed both ethics board review and privacy impact assessment. InStride adheres to the following policy guidelines: Nova Scotia Personal Health Information Act, Policy CH 07–015 Access, Use and Disclosure of Personal Health Information, and Policy CH 30 -100 Privacy and Confidentiality of Personal Health Information. InStride patient user profiles will contain only the participants’ unique study number and their baseline walk patterns. Also stored with their profile is the history of saved walking patterns as well as the unprocessed triaxial accelerometer data. These profiles are securely stored on an Azure server in Montreal Canada with SSL encryption security and access through password-controlled web portal at www.instrideweb.com. Only the patient themselves and their authorized clinical team can access their InStride profile. Each clinical team is established with secure access only to the patients they onboard to InStride. The University of Exeter accelerometer data will be stored on password protected University of Exeter computers with all personal identifiers removed and only the study number used for identification. When the data is sent for statistical analysis, all personal identifiers will be removed and the study number only used for identification. Personal identifiers (patient name, hospital number and date of birth) will be used when information about the CT scan and X-ray findings are sent to LifeIMAGE and Stryker US. At the end of the study (see timescales above), all research data will be archived as per our sponsors archiving SOP. Radiographs and scans will remain on the secure hospital systems as per routine care.
