# Supplementary material for: Alternative alignment clinical study: study protocol for a prospective, randomised, controlled study of the outcomes and cost effectiveness of triathlon CR knee replacement system: traditional philosophy versus alternative alignment philosophy
Source: J Orthop Surg Res. 2025 Dec 5;21:27. doi: 10.1186/s13018-025-06551-z (PMC12797535; doi:10.1186/s13018-025-06551-z)
Supplement: Supplementary file 1 — Supplementary Material 1 [file 13018_2025_6551_MOESM1_ESM.docx]

**MAKO Robot Assisted Left/Right Triathlon CR Total Knee Replacement**

| **Indications:** | with  involvement.  Pre op discussion of risks and benefits – including specifically DVT / PE / MI / stroke and infection / # / loosening / nerve and vessel damage / delayed wound healing / revision. |
| --- | --- |
| **Findings:** | Alignment and range of motion as recorded in appended data sheet |
| **Comorbidities:** |  |
| **Anaesthetic:** |  |
| **Procedure:** | MAKO Plan devised pre-operatively from CT scans with MPS      .  On induction IV Abx  On induction IV Tranexamic Acid  Time out performed. Routine sterile prep and drape. No tourniquet.  **Approach** –  incision.  approach.  synovitis. Bone quality .  Patella **.**  fat pad excision.  **Trackers & Planning** – Tibial tracker pins . Femoral tracker pins . Checkpoints inserted in tibia and femur. Positioning verified with robot. Peripheral osteophytes were then removed. Measurements and balancing performed and plan fine tuned, details recorded in appended data sheet. Tibial rotation .  **Bone Preparation** – Prepared as per MAKO plan to accommodate size       femoral implant and size       tibial implant. Posterior osteophytes addressed as required.  *Knee balancer:* Extension gap:      mm with      ° tension towards  side  Flexion gap:      mm,      ° tension towards  side  **Trial implants** – overall alignment  with  using   mm poly.  **Patella** – Patellar thickness   mm. Standard patella resection, resurfaced using size       button, patella height restored to   mm.  Final tibial keel preparation performed.  **Implantation** – All bone surfaces prepared with pulse lavage and dried. Chlorhex wash. Suction needle technique. of  cement pressurised with . Implants inserted without difficulty according to sizes stated above. Excess cement cleared.  **Final Check** – ML balance and ROM as recorded in appended data sheet.  Checkpoints and tracker pins removed. Haemostasis ensured. Closure in layers,  Sign out performed. Sterile dressing, wool and crepe ankle to thigh.  **Comments:** |
| Post-op **Instructions:** | Please refer to TKA Enhanced Recovery Programme guidelines in HUB  Routine and neurovascular observations  Analgesia. .  VTE Prophylaxis:  - Foot pumps in bed until mobilising well  - Early mobilisation where safe  till discharge.  - Discharge with for duration.  Day 0  Static Quadricep exercise.  No Pillow under the knee.  Commence progressive ROM and quadriceps exercise.  Consider Day 0 mobilisation FWB with frame / crutches if patient condition amenable.  Day 1  If urinary catheter in situ remove early AM  Remove bandaging.  Post-op check bloods and X-ray.  Mobilise FWB with crutches.  Continue progressive ROM and quadriceps exercise.  Discharge can occur if patient condition amenable and physios happy.  Day 2  Discharge if patient condition amenable and physios happy.  Day 3  Discharge if patient condition amenable and physios happy.  If discharge > Day 3 please email [rde-tr.ekru@nhs.net](mailto:rde-tr.ekru@nhs.net) with patient details.    Follow-up clinic – 6/52. |

**LEFT** **MAKO Data Points:**

**[TAKE SCREENSHOT OF PREPLAN SCREEN]**

**Coronal:**       °

**Sagittal:** Best extension:       ° Deepest flexion:      °

**Pre-resection soft tissue envelope:**

In Extension: On varus stress      ° On valgus stress      °

At 90: On varus stress      ° On valgus stress      °

**Estimated cartilage loss:** MFC      mm, LFC      mm, MTP      mm, LTP      mm.

| **Initial Plan** |  | Stressed Gaps: | | |  | Planned Cuts: | | |  |  |
| --- | --- | --- | --- | --- | --- | --- | --- | --- | --- | --- |
|  |  |  | **Med** | **Lat** |  |  | **Med** | **Lat** |  | **TAKE SCREENSHOT** |
|  |  | **Ext** | mm | mm |  | **DF** | mm | mm |  |  |
|  |  | **Flex** | mm | mm |  | **PF** | mm | mm |  |  |
|  |  |  |  |  |  | **T** | mm | mm |  |  |

**Adjustment Actions:**

| **Adjusted Plan** | | Planned Gaps: | | |  | |  | | | | | |  | |  | |  |
| --- | --- | --- | --- | --- | --- | --- | --- | --- | --- | --- | --- | --- | --- | --- | --- | --- | --- |
|  |  |  | **Med** | **Lat** | | | | |  | |  | |  | | **TAKE SCREENSHOT** | |  |
|  |  | **Ext** | mm | mm | | | | |  | |  | |  | |  |  |  |
|  |  | **Flex** | mm | mm | | | | |  | |  | |  | |  |  |  |
|  |  |  |  |  | |  | |  | |  | |  | |  | |  | |

**Trial Alignment:**

Coronal Femur       ° Tibia       °

Sagittal Femur      ° Tibia Slope      °

Rotation Femur TEA      ° PCA      °

| **FINAL GAPS** | | |
| --- | --- | --- |
| Gaps | Med | Lat |
| Ext | mm | mm |
| Flex | mm | mm |

**Final Alignment: Coronal:**      °correctible to      °

**Sagittal:**      °

Drop test with patella in place      °flexion

**Alignment Study Key Information**

**Key Inclusion Criteria:**

- Patient in whom any varus deformity present is <20°
- The diagnosis is of tricompartmental osteoarthritis of the knee
- Patient has primary diagnosis of Non-Inflammatory Degenerative Joint Disease
- BMI <40
- Aged = 60 – 80

**Key Exclusion Criteria:**

- If the knee for surgery has a fixed flexion deformity ≥15°
- If the knee for surgery has a varus deformity ≥20°
- If the knee for surgery has a valgus deformity i.e. HKA <0°
- Pre-op Oxford Knee Score <8°
- Pre-op knee flexion ability <90°
- Patient has a Body Mass Index (BMI) ≥ 40.
- Patient requires bilateral total knee replacements, or has a history of unsuccessful contralateral partial or total knee replacement.
- Patient has chronic heart failure (NYHA Stage ≥ 2)
- Patient has a neuromuscular or neurosensory deficiency, which limits the ability to evaluate the safety and efficacy of the device.
- Patient is diagnosed with a systemic disease (e.g. Lupus Erythematosus) or a metabolic disorder (e.g. Paget’s disease) leading to progressive bone deterioration.
- Patient is immunologically suppressed or receiving steroids in excess of normal physiological requirements (e.g. > 30 days).
- Patient requires revision surgery of a previously implanted total knee replacement or knee fusion to the affected joint.

**Triathlon CR**

**Mechanical Alignment Group**

Measured Resection workflow but in lig balancing

Coronal Femur = 0° Posterior Femur ER off PCA= 3° Femoral Flexion 0-5°

Coronal Tibia = 0° Sagittal Tibia Slope = 3° Overall Alignment HKA = 0°

6mm off non worn side

Use Ligament releases to achieve equal gaps **record releases done and grade of release 1-3

Femoral flexion + Tibial slope should not exceed 8°

**Individualised Alignment Group**

**Clear Osteophytes first**

Distal Femur 6mm cut off medial and lateral

Posterior Femur 6mm cut off medial and lateral (avoid IR or ER >5)

**If bone loss make an estimation and reduce cut

7mm cut off medial tibia, adjust lateral to get balanced gaps

**If bone loss make an estimation and reduce cut by equal amount

Slope = 0-5^0^ to balance flex / ext and to recreate **constitutional slope**

Recut tibia to balance gaps if necessary

Aim for balanced gaps

Femoral flexion + Tibial slope should not exceed 12^0^

Tibial slope alone should not exceed 7°

**Gaps**

**Lateral should never be tighter than medial.**

**Extension gap tolerance 1mm**

**Flexion gap tolerance 2mm**
